# Identification of risk factors for in-hospital death of COVID - 19 pneumonia -- lessions from the early outbreak

**DOI:** 10.1186/s12879-021-05814-4

**Published:** 2021-01-25

**Authors:** Zhigang Wang, Zhiqiang Wang

**Affiliations:** 1 Department of Cardio-thoracic Surgery, Nanjing Drum Tower Hospital, The Affiliated Hospital of Nanjing University Medical School, Nanjing, 210008 China; 2 Department of Obstetrics and Gynecology, The Central Hospital of Wuhan, Wuhan, 430014 China

**Keywords:** Characteristics, COVID-19, Risk factors, Therapy, Survivors

## Abstract

**Background:**

To examine the clinical characteristics and identify independent risk factors for in-hospital mortality of 2019 novel coronavirus (COVID-19) pneumonia.

**Methods:**

A total of 156 patients diagnosed with COVID-19 pneumonia at the Central Hospital of Wuhan from January 29, 2020, to March 20, 2020, and 20 healthy individuals were enrolled in this single-centered retrospective study. The epidemiological parameters, clinical presentations, underlying diseases, laboratory test results, and disease outcomes were collected and analyzed.

**Results:**

The median age of all enrolled patients was 66 years. At least one underlying disease was identified in 101 COVID-19 patients, with hypertension being the most common one, followed by cardiovascular disease and diabetes. The most common symptoms identified upon admission were fever, cough, dyspnea, and fatigue. Compared to survival cases, patients who died during hospitalization had higher plasma levels of D-dimer, creatinine, creatine kinase, lactate dehydrogenase, lactate, and lower percentage of lymphocytes (LYM [%]), platelet count and albumin levels. Most enrolled patients received antibiotics and anti-viral treatment. In addition, 60 patients received corticosteroids, and 51 received intravenous immunoglobulin infusion. Forty-four patients received noninvasive ventilation and 19 received invasive ventilation. Respiratory failure was the most frequently observed complication (106 [67.9%]), followed by sepsis (103 [66.0%]), acute respiratory distress syndrome (ARDS) (67 [42.9%]), and septic shock (50 [32.1%]).

Multivariable regression suggested that advanced age (OR [odds ratio] = 1.098, 95% CI [confidence interval]: 1.006–1.199, *P* = 0.037), shorter duration from onset to admission (OR = 0.853, 95% CI: 0.750–0.969, *P* = 0.015) and elevated lactate level upon admission (OR = 2.689, 95% CI: 1.044–6.926, *P* = 0.040) were independent risk factors for in-hospital mortality for COVID-19 infection. Meanwhile, increased LYM (%) at admission (OR = 0.787, 95% CI: 0.686–0.903, *P* = 0.001) indicated a better prognosis.

**Conclusions:**

In this study, we discovered that age, duration from onset to admission, LYM (%), and lactate level upon admission were independent factors that affecting the in-hospital mortality rate.

## Background

Since it was first identified, the 2019 novel coronavirus (COVID-19) has relentlessly spread all over the world and infected almost 42 million people as of Oct 23, 2020, and taken more than 1.1 million lives [1, 2]. The COVID-19 is the seventh member of the coronavirus family [3]. Compared with the 10% fatal rate in SARS-CoV infection [4] and 37% in MERS-CoV infection [5], the mortality rate of COVID-19 seems to be lower and has been estimated around 1–5% [2]. However, COVID-19 has a higher reproduction number (RO) therefore is much more contagious than its precursors, which results in an enormous burden to global health. The clinical symptoms of COVID-19 infection are generally mild, and most patients have a good prognosis. However, the conditions can deteriorate in about 10–20% of all patients who are often required to be transferred to intensive care unit (ICU) and suffered from a very high mortality rate [6].

Due to its latent onset, it is important to early identify patients with increased risks of disease progression so clinical treatment can be adjusted before disease progression. Even though numerous reports have been published about COVID-19, studies that focus on identifying such risk factors are still needed. Here in this retrospective analysis, we identified several risk factors that associated with death in COVID-19 patients and assessed the effectiveness of current treatment strategies.

## Methods

A total of 156 patients diagnosed with COVID-19 at the Central Hospital of Wuhan from January 29, 2020, to March 20, 2020, and 20 healthy individuals (age/sex matched) were included in this single-centered retrospective study. Epidemiology parameters, clinical presentations, laboratory results, and disease outcomes of enrolled patients were collected and reviewed for COVID-19 patients by two independent designated researchers. While in the control group, only laboratory results were collected.

The diagnosis was made following the Chinese COVID-19 management guideline (7th Edition) [7], and the disease severity was characterized as mild, moderate, severe, or critical according to the same guideline. More specifically, the diagnosis for the suspected patients required confirmational real-time PCR tests for COVID-19 nucleic acid with samples obtained from patients’ throat swabs and/or bronchoalveolar lavage fluid (BALF).

For severe and critical patients, corticosteroid therapy (methylprednisolone 40–120 mg per day) was given immediately upon admission or within the first 3 days of hospitalization. Antibiotics and Oseltamivir were empirically applied to all patients. Oxygen support (nasal cannula, high oxygen flow, noninvasive assisted ventilation, and mechanical ventilation) was applied to patients as needed.

### Definitions

Fever was defined as an axillary temperature above 37.3 °C. Sepsis and septic shock were defined according to the 2016 Third International Consensus Definition for Sepsis and Septic Shock [8]. Acute respiratory distress syndrome (ARDS) was diagnosed according to the Berlin Definition [9]. Acute kidney injury was identified according to the Kidney Disease: Improving Global Outcomes definition (KDIGO) guideline [10]. The acute cardiac injury was diagnosed if serum levels of cardiac enzymes increased above the upper limit of the normal value or new abnormalities identified in electrocardiography and echocardiography [6].

Discharge criteria was defined as: body temperature returned to normal and maintained at least three consecutive days; remission of respiratory symptoms; significant improvement on chest computed tomography (CT) scans and negative results on RNA tests on nasopharyngeal swabs obtained at least 24 h apart.

### Statistical analysis

Data was analyzed with SPSS software for windows (version 25.0 IBM Corp, Armonk, NY). Continuous variables were expressed as median and interquartile (IQR). The student *t-*test was applied for normally distributed continuous variables and the Mann-Whitney *U* test for nonnormally distributed variables. Categorical variables were compared with the χ^2^ test or Fisher exact test.

All parameters that might affect in-hospital mortality were screened by univariable analyses. The variables that reached significance were further tested by multivariable stepwise logistic regression analyses (stepwise enter method). After the risk factors were determined, Kaplan-Meier survival curves were plotted to reveal the effect of laboratory risk factors on in-hospital mortality. Receiver-operating characteristic (ROC) curves were constructed to assess the diagnostic value of the laboratory test results. The optimal cutoff was first assessed by You-den’s index (J = Sensitivity + Specificity − 1). STATA statistical analysis software was used to assess the difference between the areas under the receiver-operating characteristic curve (AUC). For laboratory results, we considered the normal ranges used in the Central Hospital of Wuhan as the reference. *P* < 0.05 was considered statistically significant.

## Results

The cohort included in this study consisted of 156 hospitalized patients diagnosed with COVID-19 pneumonia and 20 healthy controls. Among all patients, 56 died during hospitalization and 100 were successfully discharged. The median age of all enrolled patients was 66 years (IQR, 46.3–73.0; range, 9–99 years), and 76 (48.7%) were male. Comorbidities were identified in 101 (64.7%) patients, with hypertension, cardiovascular disease, and diabetes being the most common ones. The most common symptoms presented upon admission were fever (79 [50.6%]) and cough (71 [45.5%]), followed by dyspnea (28 [17.9%]) and fatigue (23 [14.7%]) (Table 1).

**Table 1:** Baseline characteristics of patients infected with COVID-19

	Total (*n* = 156)	Survivor (*n* = 100)	Non-survivor (*n* = 56)	*P* value^a^
**Characteristics**				
Age, years	66.0 (46.3–73.0)	58.0 (37.0–69.0)	72.0 (65.3–83.0)	**< 0.001**
Male, %	76 (48.7)	44(44.0)	32(57.1)	0.115
**Underlying disease**				
Cardiovascular disease, %	44 (28.2)	17 (17.0)	27 (48.2)	**< 0.001**
COPD, %	27 (17.3)	12 (12.0)	15 (26.8)	**0.019**
Chronic renal disease, %	15 (9.6)	4 (4.0)	11 (19.6)	**0.001**
Chronic liver disease, %	14 (9.0)	2 (2.0)	12 (21.4)	**< 0.001**
Cerebrovascular, %	22 (14.1)	9 (9.0)	13 (23.2)	**0.014**
Diabetes, %	31 (19.9)	11 (11.0)	20 (35.7)	**< 0.001**
Hypertension, %	65 (41.7)	31 (31.0)	34 (60.7)	**< 0.001**
Cancer, %	14 (9.0)	3 (3.0)	11 (19.6)	**< 0.001**
**Initial symptoms**				
Fever, %	79 (50.6)	56 (56.0)	23 (41.4)	0.074
Cough, %	71 (45.5)	45 (45.0)	26 (46.4)	0.864
Chest tightness, %	28 (17.9)	16 (16.0)	12 (21.4)	0.397
Asthma, %	30 (19.2)	22 (22.0)	8 (14.3)	0.241
Headache, %	5 (3.2)	2 (2.0)	3 (5.4)	0.351
Myalgia, %	2 (1.3)	1 (1.0)	1 (1.8)	1.000
Chill, %	4 (2.6)	4 (4.0)	0 (0)	0.297
Nausea or vomiting, %	8 (5.1)	3 (3.0)	5 (8.9)	0.136
Fatigue, %	23 (14.7)	8 (8.0)	15 (26.8)	**0.002**
Diarrhea, %	2 (1.3)	1 (1.0)	1 (1.8)	0.359
Poor appetite, %	13 (8.3)	4 (4.0)	9 (16.1)	**0.014**
Disturbance of consciousness, %	4 (2.6)	0 (0)	4 (7.1)	**0.015**

Notes: Data presented as n (%) or median (IQR)

*Abbreviations*: *COVID-19* Coronavirus Disease 2019, *IQR* interquartile range, *COPD* Chronic obstructive pulmonary disease

^a^ *P* values indicate differences between survivors and non-survivors. *P* < 0.05 was considered statistically significant

Compared with successfully discharged patients, the patients who died during hospitalization were older (72.0 years [65.3–83.0] VS 58.0 years [37.0–69.0]), and were more likely complicated with underlying diseases such as hypertension (34 [60.7%] vs 31 [31.0%]), diabetes (20 [35.7%] vs 11 [11.0%]), cardiovascular disease (27 [48.2%] vs 17 [17.0%]), cerebrovascular disease (13 [23.2%] vs 9 [9.0%]), chronic obstructive pulmonary disease (COPD) (15 [26.8%] vs 12 [12.0%]), cancer (11 [19.6%] vs 3 [3.0%]), chronic renal disease (11 [19.6%] vs 4 [4.0%]), and chronic liver disease (12 [21.4%] vs 2 [2.0%]). Compared with survivors, non-survivors were more likely to present with fatigue (15 [26.8%] vs 8 [8.0%]), anorexia (9 [16.1%] vs 4 [4.0%]), and neuropsychic symptoms (4 [7.1%] vs 0) (Table 1).

In terms of laboratory tests, multiple differences between survivors and non-survivors were identified and summarized in Table 2. Specifically, non-survivors had an increased level of white blood cell (WBC) count, neutrophil count, percentage of neutrophils, D-dimer, creatinine, creatine kinase (CK), and lactate dehydrogenase (LDH), as well as higher levels of c-reactive protein (CRP), procalcitonin (PCT) and interleukin-6 (IL-6). Whereas, percentage of lymphocytes, platelet count, and albumin levels were significantly lower in non-survivors. In addition, non-survivors had elevated levels of lactate and glucose, accompanied by lower levels of PaO_2_/FiO_2_ (Table 3). Furthermore, our result suggested that the level of LYM (%) in COVID-19 patients upon admission was significantly lower than that in the control group. In contrast, the levels of CRP, D-dimer, and lactate were higher in COVID-19 patients (Table 4).

**Table 2:** Laboratory findings of patients infected with COVID-19 on admission to hospital

	Total (*n* = 156)	Survivor (*n* = 100)	Non-survivor (*n* = 56)	*P* value^a^
WBC count, 10^9^/L	6.2 (4.7–8.3)	5.6 (4.5–7.2)	7.8 (5.5–12.6)	**< 0.001**
Neutrophil count, 10^9^/L	4.0 (3.0–6.8)	3.4 (2.5–5.0)	6.8 (4.5–11.3)	**< 0.001**
Lymphocyte count, 10^9^/L	1.2 (0.7–1.7)	1.4 (1.0–1.9)	0.7 (0.5–1.1)	0.503
NEU (%), %	71.0 (58.9–84.4)	62.9 (55.3–72.4)	86.7 (76.3–91.0)	**< 0.001**
LYM (%), %	19.7 (10.5–30.6)	27.6 (18.4–33.5)	8.7 (4.7–14.3)	**< 0.001**
Hemoglobin, g/L	123.7 (114.2–136.6)	125.0 (115.5–136.3)	122.4 (113.9–135.8)	0.246
Platelet, 10^9^/L	194.0 (157.0–249.0)	218.5 (172.3–259.5)	168.0 (114.0–200.0)	**< 0.001**
Total bilirubin, mmol/L	10.9 (7.5–17.2)	10.7 (7.5–14.6)	13.0 (7.5–25.6)	0.057
LDH, U/L	197.0 (159.5–279.0)	175.0 (149.0–219.0)	310.5 (201.0–479.3)	**< 0.001**
ALT, U/L	20.2 (13.5–39.5)	19.5 (13.0–37.9)	22.1 (14.7–41.5)	0.400
AST, U/L	21.7 (16.1–34.2)	18.8 (15.1–26.6)	30.0 (21.3–55.3)	**0.002**
Albumin, g/L	37.5 (33.8–42.6)	39.9 (36.5–43.3)	33.1 (29.7–33.6)	**< 0.001**
Globulin, g/L	28.7 (24.4–32.9)	28.2 (24.1–30.9)	31.5 (24.8–34.9)	**0.014**
BUN, mmol/L	4.7 (3.7–6.2)	4.2 (3.3–5.3)	6.2 (5.0–10.8)	**< 0.001**
Creatinine, μmol/L	66.1 (50.3–84.2)	64.8 (50.9–75.1)	74.2 (47.0–126.9)	**0.008**
CK, U/L	68.0 (45.0–121.0)	63.0 (40.0–96.0)	112.8 (62.3–245.0)	**0.028**
CK-MB, U/L	1.6 (0.8–4.6)	0.9 (0.7–1.4)	4.3 (1.7–13.3)	**0.044**
troponin I, pg/ml	20.0 (4.1–57.5)	10.0 (3.0–20.0)	50.0 (22.3–115.0)	**0.013**
BNP, ng/L	118.5 (32.5–392.7)	56.8 (20.0–132.9)	374.7 (135.1–814.5)	0.069
D-dimer, mg/L	1.0 (0.4–4.6)	0.7 (0.2–1.6)	3.3 (1.2–7.8)	**0.025**
CRP, mg/L	0.9 (0.1–3.6)	0.2 (0.1–1.6)	4.1 (2.5–7.2)	**< 0.001**
Procalcitonin, ng/mL	0.06 (0.04–0.14)	0.05 (0.04–0.06)	0.37 (0.12–0.77)	**0.022**
IL-6, pg/mL	7.1 (2.4–24.7)	2.9 (1.5–7.4)	79.6 (9.6–212.5)	**0.027**
CD19+, count/μL	12.6 (9.0–18.9)	11.1 (8.6–17.0)	16.6 (10.1–19.1)	0.292
CD3+, count/μL	68.9 (57.4–75.3)	70.6 (62.0–76.7)	66.8 (54.6–71.4)	0.329
CD4+, count/μL	38.8 (34.6–46.0)	38.8 (31.4–45.2)	39.4 (36.2–52.5)	0.183
CD8+, count/μL	25.3 (19.2–32.3)	26.7 (19.6–33.9)	20.5 (13.9–30.5)	0.063
CD4/CD8	1.6 (1.1–2.3)	1.5 (1.0–2.1)	1.8 (1.2–3.9)	0.071

Notes: Data presented as n (%) or median (IQR)

*Abbreviations*: *COVID-19* Coronavirus Disease 2019, *WBC* White blood cell, *NEU (%)* Percentage of neutrophils, *LYM (%)* Percentage of lymphocytes, *LDH* Lactate dehydrogenase, *ALT* Alanine aminotransferase, *AST* Aspartate aminotransferase, *BUN* Blood urea nitrogen, *CK* Creatine kinase-MB, *CK-MB* Creatine kinase-MB, *BNP* Brain natriuretic peptide, *CRP* C-reactive protein, *IL-6*,Interleukin-6

^a^*P* values indicate differences between survivors and non-survivors. *P* < 0.05 was considered statistically significant

**Table 3:** Blood gas analysis of patients infected with COVID-19

	Total (*n* = 156)	Survivor (*n* = 100)	Non-survivor (*n* = 56)	*P* value^a^
Ph	7.44 (7.39–7.47)	7.43 (7.40–7.46)	7.45 (7.39–7.48)	0.970
PaO_2_, mm Hg	91.0 (64.3–119.0)	95.0 (79.0–129.0)	72.0 (50.0–116.0)	**0.049**
PaO_2_/FiO_2_, mm Hg	195.0 (90.0–262.5)	225.0 (152.5–287.5)	117.5 (78.3–192.9)	**< 0.001**
PaCO_2_, mm Hg	38.0 (34.0–42.0)	40.0 (35.0–45.0)	36.0 (33.0–40.0)	0.113
BE, mmol/L	1.9 (−0.7–4.1)	2.1 (− 0.1–3.9)	1.2 (−1.8–4.6)	0.300
K^+^, mmol/L	3.8 (3.4–4.1)	3.8 (3.5–4.2)	3.8 (3.3–4.1)	0.768
Na^+^, mmol/L	140.0 (136.0–143.8)	141.0 (138.0–144.0)	137.0 (133.0–142.0)	0.641
Ca^2+^, mmol/L	0.9 (0.7–1.0)	0.9 (0.7–1.1)	0.9 (0.7–1.0)	0.482
Lactate, mmol/L	1.7 (1.2–2.3)	1.5 (1.1–2.1)	2.0 (1.5–2.9)	**0.005**
Hematocrit, %	37.0 (32.0–41.0)	38.0 (32.5–42.0)	36.0 (30.0–40.0)	0.627
Glucose, mmol/L	6.7 (5.4–8.7)	5.9 (4.9–7.9)	7.4 (6.2–11.1)	**0.034**

Notes: Data presented as median (IQR)

*Abbreviations*: *COVID-19* Coronavirus Disease 2019, *PaO*_*2*_ Partial pressure of oxygen, *PaO*_*2*_ Partial pressure of carbon dioxide, *BE* Base excess

^a^*P* values indicate differences between survivors and non-survivors. *P* < 0.05 was considered statistically significant

**Table 4:** Comparision of the laboratory levels between the COVID-19 and healthy control group

	Covid-19 group (*n* = 156)	Control group (*n* = 20)	*P* value
Age, years	66.0 (46.3–73.0)	66.0 (44.8–73.3)	0.968
Male, %	76 (48.7)	10 (50)	0.886
WBC count, 10^9^/L	6.2 (4.7–8.3)	5.6 (4.0–7.0)	0.090
LYM (%), %	19.7 (10.5–30.6)	27.2 (22.2–34.1)	**0.003**
Creatinine, μmol/L	66.1 (50.3–84.2)	67.8 (53.5–75.0)	0.891
CRP, mg/L	0.9 (0.1–3.6)	0.4 (0.1–0.6)	**0.016**
D-dimer, mg/L	1.0 (0.4–4.6)	0.4 (0.2–0.7)	**< 0.001**
Lactate, mmol/L	1.7 (1.2–2.3)	1.2 (0.8–1.4)	**< 0.001**

Notes: Data presented as n (%) or median (IQR)

*Abbreviations*: *COVID-19* Coronavirus Disease 2019, *WBC* White blood cell, *LYM (%)* Percentage of lymphocytes, *CRP* C-reactive protein

*P* < 0.05 was considered statistically significant

150 (96.2%) patients received antibiotics and 139 (89.1%) received antiviral treatment. Unsurprisingly, systematic corticosteroid was more commonly applied in non-survivors. Fifty-six patients (35.9%) received high-flow nasal cannula oxygen therapy, 44 (28.2%) received non-invasive mechanical ventilation and 19 patients (12.2%) required invasive mechanical ventilation support. Six patients (3.8%) received renal replacement therapy (RRT) and no patients were treated with extracorporeal membrane oxygenation therapy. Oxygen support (including high oxygen flow, noninvasive assisted ventilation, and mechanical ventilation) and renal replacement therapy was more commonly applied in non-survivors (Table 5).

**Table 5:** Treatments of patients infected with COVID-19

	Total (*n* = 156)	Survivor (*n* = 100)	Non-survivor (*n* = 56)	*P* value^a^
Antibiotics, %	150 (96.2)	96 (96.0)	54 (96.4)	1.000
Antiviral treatment, %	139 (89.1)	89 (89.0)	50 (89.3)	0.956
Corticosteroids, %	60 (38.5)	22 (22.0)	38 (67.9)	**< 0.001**
Intravenous immunoglobulin, %	51 (32.7)	18 (18.0)	33 (58.9)	**< 0.001**
High-flow nasal cannula oxygen therapy, %	56 (35.9)	26 (26.0)	30 (53.6)	**0.001**
Non-invasive mechanical ventilation, %	44 (28.2)	13 (13.0)	31 (55.4)	**< 0.001**
Invasive mechanical ventilation, %	19 (12.2)	1 (1.0)	18 (32.1)	**< 0.001**
RRT, %	6 (3.8)	1 (1.0)	5 (8.9)	**0.023**

Notes: Data presented as n (%)

*Abbreviations*: *COVID-19* Coronavirus Disease 2019, *RRT* Renal replacement therapy

^a^*P* values indicate differences between survivors and non-survivors. *P* < 0.05 was considered statistically significant

The clinical outcomes of the enrolled 156 COVID-19 patients were summarized in Table 6. Unsurprisingly, non-survivor patients were more likely to develop complications compared with survivors. Respiratory failure was the most frequently developed complication (106 [67.9%]), followed by sepsis (103 [66.0%]), ARDS (67 [42.9%]), septic shock (50 [32.1%]), arrhythmia (42 [26.9%]), acute cardiac injury (26 [16.7%]), cardiac failure (24 [15.4%]), and acute kidney injury (18 [11.5%]). The median time from disease onset to admission was 10.0 days (IQR 4.3–16.0). And the median time from disease onset to discharge was 36.0 days (IQR 27.3–48.0), whereas the median time to death was 17.0 days (IQR 10.0–26.5). After comparing the time from disease onset to hospital admission and total hospital stay between non-survivors with survivors [(median time, 6.0 days [1.0–10.0] vs 14.5 days [7.0–20.0]) and (median time, 9.0 days [3.3–16.0] vs 22.0 days [16.0–29.0]) respectively], it seemed like the disease progressed more rapidly in non-survivors.

**Table 6:** Outcomes of patients infected with COVID-19

	Total (*n* = 156)	Survivor (*n* = 100)	Non-survivor (*n* = 56)	*P* value^a^
Arrhythmia, %	42 (26.9)	13 (13.0)	29 (51.8)	**< 0.001**
Sepsis, %	103 (66.0)	49 (49.0)	54 (96.4)	**< 0.001**
ARDS, %	67 (42.9)	17 (17.0)	50 (89.3)	**< 0.001**
Respiratory failure, %	106 (67.9)	50 (50.0)	56 (100)	**< 0.001**
Cardiac failure, %	24 (15.4)	11 (11.0)	13 (35.9)	**0.043**
Septic shock, %	50 (32.1)	9 (9.0)	41 (73.2)	**< 0.001**
Acute kidney injury, %	23 (14.7)	8 (8.0)	15 (26.8)	**0.002**
Acute cardiac injury, %	26 (16.7)	6 (6.0)	20 (35.7)	**< 0.001**
Onset to admission, days	10.0 (4.3–16.0)	14.5 (7.0–20.0)	6.0 (1.0–10.0)	**< 0.001**
Hospitalization, days	18.0 (11.0–27.8)	22.0 (16.0–29.0)	9.0 (3.3–16.0)	**< 0.001**
Onset to discharge or death, days	30.0 (21.0–42.8)	36.0 (27.3–48.0)	17.0 (10.0–26.5)	**< 0.001**

Notes: Data presented as n (%) or median (IQR)

*Abbreviations*: *COVID-19* Coronavirus Disease 2019, *ARDS* Acute respiratory distress syndrome

^a^*P* values indicate differences between survivors and non-survivors. *P* < 0.05 was considered statistically significant

Next, multivariable logistic regression assay discovered that age (OR [odds ratio] = 1.098, 95% CI [confidence interval]: 1.006–1.199, *P* = 0.037), duration from onset to admission (OR = 0.853, 95% CI: 0.750–0.969, *P* = 0.015), LYM (%) at admission (OR = 0.787, 95% CI: 0.686–0.903, *P* = 0.001), and lactate at admission (OR = 2.689, 95% CI: 1.044–6.926, *P* = 0.040) were independent risk factors for in-hospital death of COVID-19 pneumonia (Table 7).

**Table 7:** Risk factors associated with in-hospital death infected with COVID-19

	Univariable Analyses			Multivariable Analyses		
	OR	95% CI	*P* value	OR	95% CI	*P* value
Age	1.083	1.051–1.117	< 0.001	1.098	1.006–1.199	**0.037**
Onset to admission	0.870	0.821–0.922	< 0.001	0.853	0.750–0.969	**0.015**
Cardiovascular disease	4.546	2.170–9.523	< 0.001	0.321	0.239–12.863	0.581
Hypertension	3.440	1.737–6.814	< 0.001	0.124	0.012–1.278	0.079
Diabetes	4.495	1.957–10.322	< 0.001	2.744	0.323–23.304	0.355
Creatinine	1.012	1.004–1.021	0.005	1.006	0.997–1.015	0.171
CRP	1.427	1.223–1.665	< 0.001	1.086	0.897–1.313	0.398
LYM (%)	0.816	0.765–0.871	< 0.001	0.787	0.686–0.903	**0.001**
D-dimer	1.047	1.001–1.095	0.047	0.987	0.941–1.036	0.599
Lactate	1.738	1.209–2.498	0.003	2.689	1.044–6.926	**0.040**
Corticosteroids	7.485	3.594–15.590	< 0.001	1.162	0.889–1.518	0.064
Immunoglobulin	6.536	3.127–13.663	< 0.001	2.896	0.771–10.877	0.115
Acute kidney injury	4.207	1.654–10.703	0.003	12.502	0.188–832.413	0.238
Acute cardiac injury	8.704	3.234–23.421	< 0.001	14.875	0.536–187.673	0.123
Cardiac failure	2.446	1.013–5.907	0.047	1.788	0.075–42.669	0.720

*Abbreviations*: *COVID-19* Coronavirus Disease 2019, *OR* Odds ratio, *CI* Confidence interval, *CRP* C-reactive protein, *LYM (%)* Percentage of lymphocytes

*P* < 0.05 was considered statistically significant

**Fig. 1:**
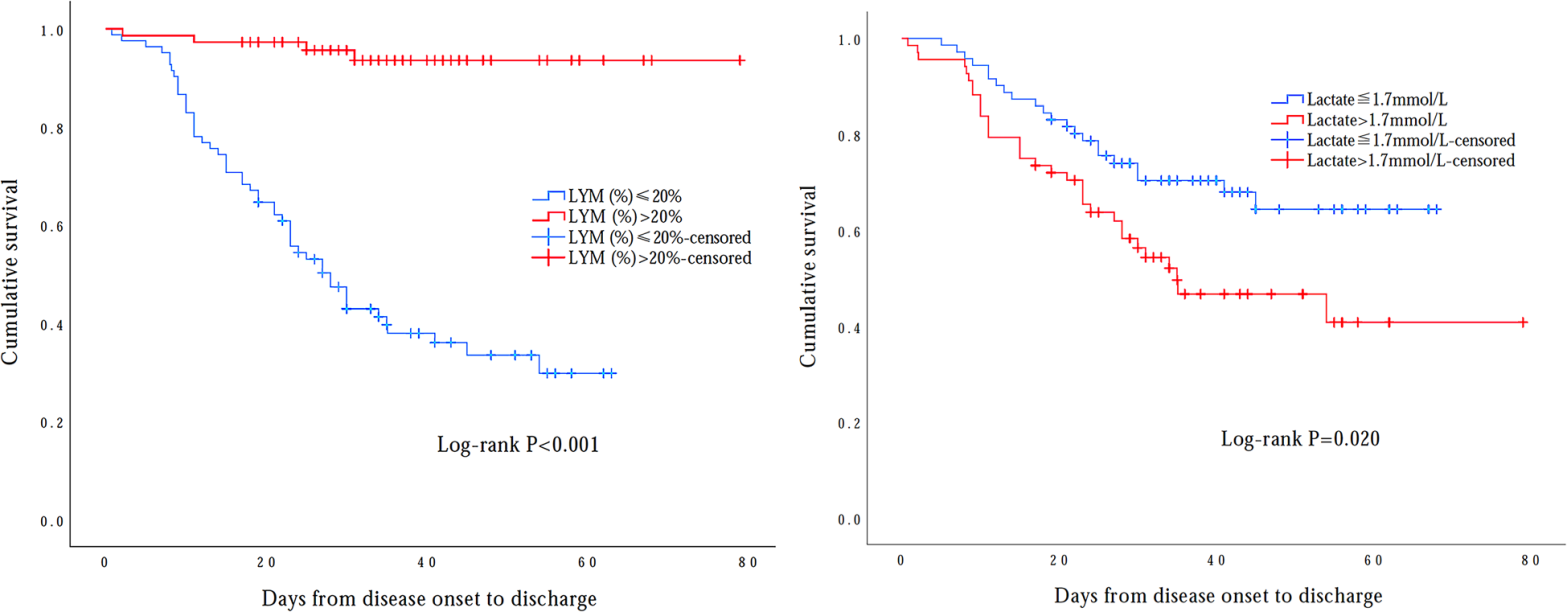
Kaplan-Meier survival curve for COVID-19 patients with different counts of LYM (%) (*P* < 0.001) and serum lactate (*P* = 0.020) (COVID-19, Coronavirus Disease 2019; LYM [%], Percentage of lymphocytes)

**Fig. 2:**
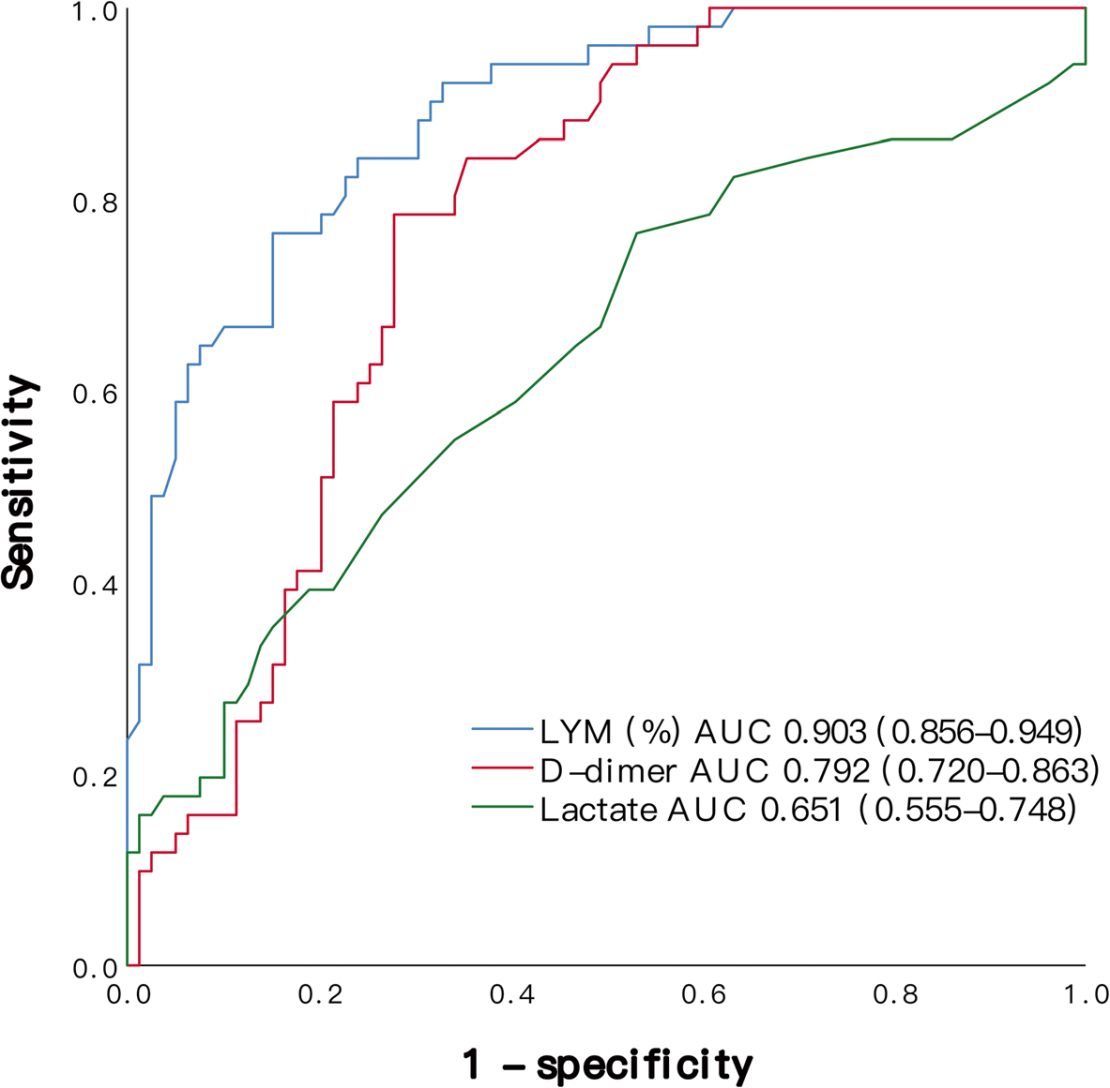
Receiver-operating characteristic of LYM (%), D-dimer and lactate upon hospital admission (AUC, Areas under receiver-operating characteristic curve; LYM[%], Percentage of lymphocytes)

The Kaplan-Meier survival curve showed a trend toward poorer survival in COVID-19 patients with increased lactate levels and/or decreased LYM (%) upon admission (*P* = 0.020 and *P* < 0.001, respectively) (Fig. 1). In addition, we conducted the ROC curve assay and calculated the AUC among the following three indicators. We discovered that the AUC of LYM (%) was 0.903 (95% CI, 0.856–0.949), 0.792 (95% CI, 0.720–0.863) for D-dimer and of 0.651 (95% CI, 0.555–0.748) for lactate (Fig. 2). Comparing to the other indicators, the AUC of LYM (%) was higher in predicting in-hospital death (LYM [%] VS D-dimer, *P* = 0.003; LYM [%] VS lactate, *P* < 0.001; respectively). Furthermore, we discovered that the cutoff value of LYM (%) for predicting in-hospital death was 14.7%.

## Discussion

In this study, we reported a cohort of 156 patients with laboratory-confirmed COVID-19 pneumonia. After carefully summarized and compared patients’ clinical characteristics, we identified several risk factors for in-hospital death. Specifically, our data suggested that advanced age, shorter duration from onset to admission, decreased LYM (%) and increased lactate at admission were associated with higher odds of in-hospital death.

The Central Hospital of Wuhan is the largest tertiary hospital around the Huanan Seafood Wholesale Market area, were speculated to be ground zero for this pandemic [11–13]. Considering the fact that most of the enrolled patients lived close to this market, our data might include a large portion of patients with so-called “primary infection”. Comparing with other reports [6, 14, 15], patients included in our cohort were older and more commonly complicated with underlying diseases. It seemed like that the patients in our study were much severer when they were diagnosed and had a longer hospital stay.

Our data suggested symptoms like fatigue, anorexia, and neuropsychic presentations were more common in the critically ill patients. The onset and persistence of these symptoms might suggest an unfavorable prognosis. Time from disease onset to admission and death was much shorter for non-survivors, which might imply a more rapid disease progression.

After carefully reviewed the medical records of all enrolled patients, we found that 26.9% of patients with COVID-19 pneumonia presented with arrhythmia and 16.7% complicated with acute cardiac injuries. Further analysis indicated that the incidence of myocardial injury was much higher in non-survivors. In Li’s report [16], they found at least 8% of patients with COVID-19 suffered an acute cardiac injury and this ratio was 12% in Wang’s research [15]. The pathogenesis of COVID-19 infection-related acute myocardial injury is still under-studied. But according to the clinical presentation and available laboratory results, we speculated that the direct assault from the virus, hypoxemia induced by pneumonia, and over-reacting immune response all play important roles in the pathogenesis.

Consistently, our study confirmed that advanced age was associated with increased mortality in patients with COVID-19, as reported by Zhou and colleagues [14]. Previously, advanced age has been identified as an important predictor of mortality in SARS and MERS infection [17, 18]. Though has not been verified in mechanism studies, several reasons may contribute to this age-related vulnerability: firstly, patients with advanced ages are usually suffered decreased cardiopulmonary compliance and reserve thus more difficulty in coping with the disequilibrium of the cardiopulmonary system induced by COVID-19 infection; secondly, previous studies indicated that advance age was associated with more robust host innate responses but decreased in cellular as well as humoral immune functions during virus infection [19, 20]; thirdly, aged patients have an increased risk of having comorbidities which have been proved in several studies associate with worse prognosis; finally, the diagnosis and treatments in patients with advanced ages are more likely to be delayed due to atypical symptoms. Thus, attention should be paid to COVID-19 patients of advanced ages, especially to whom having multiple comorbidities.

The shorter duration from disease onset to admission was an important factor highly related to odds for death in confirmed cases of COVID-19, which was contrary to previous studies [21, 22]. This might due to the lack of understanding of this disease in the early pandemic when a large portion of patients who had mild or moderate illness refused to seek proper medical treatment. The shorter time from disease onset to admission for non-survivors in our study might imply a more rapid disease progression than we expected. The etiology of susceptibility to severe lung injury remains unclear. A recent study concluded that the determinants of disease severity seem to stem mostly from host factors, whereas viral genetic variation did not significantly affect outcomes [23]. The balance between angiotensin-converting enzyme (ACE) 1 and ACE 2 activity as the host factors has been implicated in the pathogenesis of respiratory diseases and could play a role in the severity of COVID-19 [24].

The multivariable logistic regression assay suggested that the decreased LYM (%) was an independent risk factor for in-hospital death and further analysis concluded that LYM (%) was a stronger indicator in predicting in-hospital death by the ROC assay. Previous studies showed that lymphopenia was a risk factor for increased mortality rate for SARS and COVID-19 [14, 25]. While in our study, there was no statistical difference observed in terms of lymphopenia between survivors and non-survivors. Liu and colleagues [26] demonstrated that the percentage of lymphocytes (LYM [%]) was a potential predictor of COVID-19 severity. Considering the fact that the WBC counts were significantly higher in the non-survivors in our cohort which might bias the result, we substituted the absolute lymphocyte count with the LYM [%] in the regression analysis model and repeated the assay. The decreased LYM (%) might be explained by the fact that coronavirus was able to destroy lymphocytes during an acute process. The decreased LYM% may reflect an under-activation and/or over exhausting of the immune system that consequently unable to control COVID-19 infection.

Serum lactate was identified as another risk factor associated with in-hospital death in our study. Lactate has been used as a prognostic marker in predicting the severity and outcome of sepsis and septic shock [27]. Shankar-Hari et al. suggested in their study that the adjusted odds ratio for hospital mortality increased linearly with lactate levels with lactate level > 2 mmol/L being the cutoff value for the diagnosis of septic shock [28]. This finding had been further confirmed by some recent studies [29–31]. In sepsis, patients usually experience hyperlactatemia as a consequence of tissue hypoperfusion, as well as a diminished lactate clearance rate due to dysfunction of the liver and kidney [32]. Inconsistent with our study, Zhou and colleagues [14] identified that sepsis and septic shock was a major complication for COVID-19 patients. Measurement of serum lactate seems to be a simple yet effective strategy to identify patients with increased risks.

A previous study suggested that about 90% of patients with severe pneumonia had increased coagulation activity, marked by the increased D-dimer concentrations [33]. High levels of D-dimer were proved to be associated with an increased mortality rate in patients with sepsis identified in the emergency room [34]. Previous COVID-19 studies also demonstrated that D-dimer greater than 1 μg/ml was associated with poor prognosis [14]. While in our study, D-dimer was not independently associated with in-hospital death. This discrepancy might be due to the difference in patient selection. Future studies with a larger population are needed to confirm the conclusions.

### Limitations

This study had several limitations. Firstly, this study was a retrospective study conducted in a single-center, with a cohort that might not necessarily representable for the general population. Secondly, by excluding patients still in hospital receiving treatment as of March 20, 2020, the mortality rate in our study might be biased. Finally, the lack of more effective antiviral drugs and life support methods like extracorporeal membrane oxygenation in our hospital might contribute to the poor clinical outcomes in some severe patients.

## Conclusions

Our study indicated that the non-survivors of COVID-19 were older and with a disease course that progressed more rapidly compared to survivors. Advanced age, shorter duration from onset to admission, decreased LYM (%), and increased lactate level upon hospital admission were independent risk factors for in-hospital death of patients with COVID-19 during the early outbreak.

## Data Availability

The datasets used and/or analysed during the current study are available from the corresponding author on reasonable request.

## Abbreviations

COVID-19: 2019-novel coronavirus
OR: Odds ratio
CI: Confidence interval
LYM (%): Percentage of lymphocytes
ARDS: Acute respiratory distress syndrome
ROC: Receiver-operating characteristic
ICU: Intensive care unit
BALF: Bronchoalveolar lavage fluid
KDIGO: Kidney Disease: Improving Global Outcomes
CT: Computed tomography
AUC: Areas under receiver-operating characteristic curve
MERS: Middle east respiratory syndrome
COPD: Chronic obstructive pulmonary disease
WBC: White blood cell
CK: Creatine kinase
LDH: Lactate dehydrogenase
CRP: C-reactive protein
PCT: Procalcitonin
IL-6: Interleukin-6
IQR: Interquartile range
NEU (%): Percentage of neutrophils
ALT: Alanine aminotransferase
AST: Aspartate aminotransferase
BUN: Blood urea nitrogen
CK-MB: Creatine kinase-MB
BNP: Brain natriuretic peptide
PaO2: Partial pressure of oxygen
PaCO2: Partial pressure of carbon dioxide
BE: Base excess
RRT: Renal replacement therapy
